# Respiratory Pathogen Coinfection During Intersecting COVID-19 and Influenza Epidemics

**DOI:** 10.3390/pathogens13121113

**Published:** 2024-12-17

**Authors:** Lina Jiang, Yifei Jin, Jingjing Li, Rongqiu Zhang, Yidun Zhang, Hongliang Cheng, Bing Lu, Jing Zheng, Li Li, Zhongyi Wang

**Affiliations:** 1 Xiamen Center for Disease Control and Prevention, 681 Shengguang Road, Xiamen 361021, China; ln_jiang@126.com (L.J.); zhrq19861023@sina.com (R.Z.); eatonzhang@163.com (Y.Z.); zhengjing1103@foxmail.com (J.Z.); 2 National Key Laboratory of Advanced Biotechnology, Beijing Institute of Biotechnology, 20 Dongdajie Road, Beijing 100071, China; christina_jyf@foxmail.com (Y.J.); lijjamms85@126.com (J.L.); cheng107315@163.com (H.C.); 13693506666@163.com (B.L.)

**Keywords:** respiratory pathogen, coinfection, intersecting epidemics

## Abstract

Respiratory pathogen coinfections pose significant challenges to global public health, particularly regarding the intersecting epidemics of COVID-19 and influenza. This study investigated the incidences of respiratory infectious pathogens in this unique context. We collected throat swab samples from 308 patients with a fever from outpatient and emergency departments at sentinel surveillance hospitals in Xiamen, southeast of China, between April and May 2023, testing for SARS-CoV-2 and 26 other respiratory pathogens. The coinfection rate of the XBB SARS-CoV-2 variant with other respiratory pathogens was higher than that observed during the Alpha and Delta phases. Among patients with influenza, bacterial coinfections were more prevalent. Only 0.65% (2/308) of the patients were concurrently infected with both COVID-19 and influenza. Age-stratified analysis showed a clear pattern, with a higher incidence of coinfections in children under 18 years of age. These findings highlight the need for the timely detection of respiratory pathogen coinfections and for the implementation of appropriate interventions, crucial for reducing disease burden during intersecting respiratory epidemics.

## 1. Introduction

Respiratory infectious diseases, particularly those involving coinfection with multiple pathogens, present unique epidemiological and clinical challenges [1]. The timely monitoring of coinfection with respiratory pathogens is crucial for understanding the pathogen interactions within the population; this monitoring can be undertaken by revising effective epidemic prevention policies and developing effective treatment strategies. Historically, coinfection with respiratory pathogens was common during many previous infectious disease pandemics [2]. Nonpharmaceutical interventions implemented during the COVID-19 pandemic may have decreased the incidence of respiratory pathogens. However, the COVID-19 epidemic prevention and control policies were changed at the end of 2022, which might have affected not only the pandemic’s development but also coinfection with SARS-CoV-2 and other respiratory pathogens. Following the lifting of public health restrictions, respiratory virus coinfections are more likely to occur during simultaneous COVID-19 and influenza outbreaks.

Previous studies indicated that the rate of coinfection with SARS-CoV-2 and other respiratory pathogens was less than 10% [2,3]. However, a wave of COVID-19 in China in the winter of 2022 led to a sharp increase in SARS-CoV-2’s coinfection rate with other respiratory pathogens, and this coinfection might have contributed to the severity of COVID-19 [4]. In two investigations by Swets MC et al. and Zheng et al., 0.4–3.26% of the patients with COVID-19 were coinfected with the influenza virus during the early stage of the COVID-19 epidemic in 2020–2021 [5,6]. Different animal models have shown that coinfection with SARS-CoV-2 and influenza A virus (IAV) increased weight loss and inflammatory lung damage [7]. More importantly, the severity of COVID-19 and coinfection with other respiratory pathogens may have been associated with the type of SARS-CoV-2 variant contracted by patients. However, the above studies on respiratory pathogen coinfection were performed during the Alpha and Delta variant waves, and data associated with Omicron variants were rarely reported [8].

**Figure 1:**
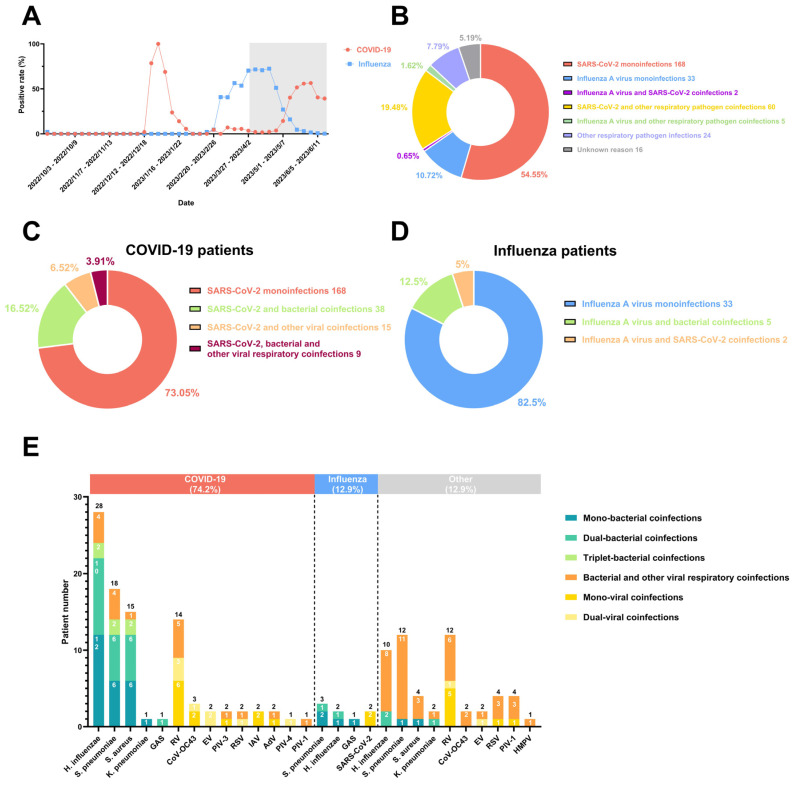
The incidence trends, coinfection types, and distributions of respiratory pathogens during the intersecting epidemics of COVID-19 and influenza: (**A**) The surveillance data for COVID-19 and influenza from multiple sentinel hospitals of Xiamen CDC in China from October 2022 to June 2023. Gray represents the times of the most recent intersecting COVID-19 and influenza epidemics in this study and throat swab collection. (**B**) The diagnostic distribution of coinfections with respiratory pathogens in 308 patients. Coinfection with other respiratory pathogens in patients with COVID-19 (**C**) or influenza (**D**). (**E**) The proportion of patients coinfected with different species of respiratory pathogens. Abbreviation: *Haemophilus influenzae* (*H. influenzae*), *Streptococcus pneumoniae* (*S. pneumoniae*), *Staphylococcus aureus* (*S. aureus*), Group A *Streptococcus* (GAS), *Klebsiella pneumoniae* (*K. pneumoniae*), Rhinovirus (RV), Coronavirus OC43 (CoV-OC43), Enterovirus (EV), Parainfluenza virus type III (PIV-3), Respiratory syncytial virus (RSV), influenza A virus (IAV), Adenovirus (AdV), Parainfluenza virus type I (PIV-1) and Parainfluenza virus type IV (PIV-4), and Human metapneumovirus (HMPV).

Between April and May 2023, the Omicron XBB subvariant epidemic peaked in southern China, while the influenza epidemic decreased (Figure 1A). The circulating XBB subvariant had higher immune evasion abilities and transmissibility than the earlier Omicron variants [9], and it may affect the condition of coinfection among respiratory pathogens. Thus, the timely monitoring of coinfection with respiratory pathogens is necessary for implementing treatment strategies. In this study, we investigated the incidence of coinfection with respiratory pathogens during the unique intersecting COVID-19 and influenza epidemics, providing new insights into the shifting epidemiology of respiratory viruses in a post-pandemic context.

## 2. Materials and Methods

### 2.1. Sample Collection

Throat swab samples were collected from 308 patients with fever within 72 h of symptom onset, and we conducted tests for SARS-CoV-2 and 26 other respiratory pathogens (Supplementary Table S1). All the patients were treated in the outpatient and emergency departments of hospitals with sentinel surveillance.

### 2.2. Pathogen Testing

The SARS-CoV-2 RNA throat swab tests were carried out with a test kit (Triplex International Biosciences Co., Ltd., Xiamen, China), and a Multiplex Real-Time PCR Diagnostic Kit (Applied Biological Technologies Co., Ltd., Beijing, China) was used to test for 26 other species of respiratory pathogens, including IAV, influenza B virus, Rhinovirus (RV), Coronavirus NL63/229E/OC43/HKU1, Parainfluenza virus types I/II/III/IV, Human metapneumovirus, Bocavirus, respiratory syncytial virus, Adenovirus, Enterovirus, *Mycoplasma pneumoniae*, *Chlamydia pneumoniae*, *Legionella pneumophila*, *Klebsiella pneumoniae*, *Bordetella pertussis*, *Haemophilus influenzae* (*H. influenzae*), *Pseudomonas aeruginosa*, Group A *Streptococcus*, *Staphylococcus aureus* (*S. aureus*), and *Streptococcus pneumoniae* (*S. pneumoniae*). The SARS-CoV-2 RNA test kit was used for the in vitro qualitative detection of the ORF1ab and N genes in the COVID-19 patients’ throat swab samples. The test kit employed for detecting 26 other species of respiratory pathogens uses real-time fluorescent PCR technology and is suitable for the nucleic acid detection of respiratory pathogens extracted from the throat swab samples.

### 2.3. Data Visualization

In this study, coinfection rates were analyzed using percentages to describe the distribution of bacterial, viral, and no coinfection cases among the 308 patients. Statistical analysis was performed using GraphPad Prism 10.1.2and Microsoft Excel for data analysis and visualization.

## 3. Results

Of the 308 patients, 74.68% (230/308) tested positive for COVID-19, 12.99% (40/308) were diagnosed with influenza, and only 0.65% (2/308) were coinfected with SARS-CoV-2 and IAV (Figure 1B). In addition, 7.79% (24/308) were infected with one or several other respiratory pathogens, and 5.19% (16/308) had no detectable cause of infection.

We conducted further analysis of coinfection conditions in the patients with COVID-19 and influenza with other respiratory pathogens. We found that 26.95% (62/230) of the COVID-19 patients were coinfected with other respiratory pathogens. Among them, 16.52% (38/230) were coinfected with bacteria, 6.52% (15/230) were coinfected with other respiratory viruses, and 3.91% (9/230) were coinfected with both bacteria and other respiratory viruses (Figure 1C). Compared with previous studies, the SARS-CoV-2–bacteria coinfection rate was comparable with that of the Omicron phase [4] but higher than those in the Alpha and Delta phases [5,8]; furthermore, the proportion of SARS-CoV-2 coinfection with other viral respiratory pathogens increased [4]. Nine rare cases of coinfection with SARS-CoV-2, bacteria, and other respiratory viruses were found. Regarding the patients with influenza, 17.5% (7/40) were coinfected with other respiratory pathogens, 12.5% (5/40) were coinfected with bacteria, and 5.0% (2/40) were coinfected with other respiratory viruses (Figure 1D). No cases of coinfection with respiratory viruses other than SARS-CoV-2 were found in the patients with influenza. Therefore, in both patients with COVID-19 and influenza, the rate of bacterial coinfection is higher than that of coinfection with other viruses, likely due to the different immune responses elicited by various pathogens.

We also analyzed the distribution of coinfected respiratory pathogens in both the patients with COVID-19 and patients with influenza (Figure 1E). The results revealed that the top three bacterial coinfections for those with COVID-19 were *H. influenzae* (28/230), *S. pneumoniae* (18/230), and *S. aureus* (15/230), while the patients with influenza were most commonly infected with *S. pneumoniae* (3/40), *H. influenzae* (2/40), and Group A *Streptococcus* (1/40). In addition, the most common viral pathogen coinfected with SARS-CoV-2 was RV (14/230), even during the influenza epidemic. For the patients with influenza, however, only 2/40 were coinfected with SARS-CoV-2, and no other coinfection virus was found.

Additionally, our study highlights the epidemiological characteristics of respiratory pathogen coinfections (Table 1). Coinfected individuals were predominantly identified in the <18 and 18–44 age groups. Among the patients with COVID-19, coinfections with *H. influenzae*, *S. pneumoniae*, *S. aureus*, and RV all occurred in over 70% of the cases within the <18 age group. Among the patients with influenza, coinfections with *H. influenzae* and *S. pneumoniae* were observed at an incidence of 50% or higher within the <18 age group. Among the patients without COVID-19 or influenza, *H. influenzae* and RV showed the highest incidences among the coinfecting pathogens, particularly in individuals under 18 years of age. Overall, respiratory pathogen coinfections exhibit a notable age-related pattern, with a higher prevalence in those under 18 years of age across all infection types, especially in COVID-19 and non-COVID-19 and non-influenza cases. This finding suggests increased susceptibility to multiple infections in children under 18 years of age.

## 4. Discussions

The coinfection rates of the COVID-19 and influenza patients with other respiratory pathogens showed a stable or increasing trend compared with previous studies, particularly with a higher prevalence of bacterial coinfections. Compared with the Alpha and Delta phases, the higher coinfection rate observed during the XBB phase could be influenced by several factors, including the higher immune evasion and transmissibility of the XBB variant, as reported in previous studies [10,11]. Furthermore, the relaxation of nonpharmaceutical interventions in late 2022 may have contributed to increased interactions between pathogens. We acknowledge that environmental and social factors during the XBB phase might have played a role, though these were not explicitly quantified in our study.

The observed age-related pattern of respiratory pathogen coinfections suggests a higher susceptibility to coinfections in children under 18 years of age. This finding could be attributed to several factors, including the immaturity of the immune system in children, increased exposure opportunities in social environments such as schools, and potentially more proactive healthcare-seeking behaviors in individuals under 18 [12,13,14]. These results highlight the importance of targeted interventions, such as vaccination and early diagnostic testing, for pediatric populations during overlapping respiratory epidemics.

While host-specific factors, such as age and immune maturity, play a critical role in determining coinfection susceptibility, the interactions between co-circulating pathogens may further influence coinfection dynamics. When multiple pathogens coexist, they may either compete or cooperate due to the pathogen–pathogen interactions [3,15]. Epidemiological negative interactions may exist between IAV and RV [16], potentially accounting for the lack of IAV and RV coinfection cases in this study. The lower coinfection rate observed (2/230) could also be attributed to the negative interactions between the IAV and SARS-CoV-2. Surprisingly, SARS-CoV-2 was not in competition with RV, or at least not the Omicron XBB subvariant.

A previous study has suggested that IAV preinfection at the cellular level significantly promotes SARS-CoV-2 (WT strain IVCAS 6.7512) infection across various cell types [17]. Moreover, more lung damage was observed in COVID-19 animal models with IAV preinfection [7]. However, there was not enough clinical evidence to support these findings based on cellular or animal models. For instance, the symptoms of the patients with COVID-19 in this study are mild. Further research is needed to gain a better understanding of the potential connection between SARS-CoV-2 and other respiratory pathogens in terms of coinfection and enhanced infectivity.

The proportion of coinfections and the types of bacteria involved in patients with COVID-19 or influenza differ between our study and other reports, potentially due to variations in the seasons and regions where the specimens were collected. The timeframe of this study includes the unique overlapping period between the peak of the Omicron XBB subvariant epidemic and the decline of the influenza epidemic. It covers outpatient and emergency cases from sentinel surveillance hospitals in Xiamen, southeast China, ensuring that the sample reflects the epidemiological trends in the region during the study period. However, the study is limited by its focus on a single city, which may be influenced by local cultural and social factors, as well as public health policies. Additionally, the inability to determine the order of multiple infections limits a more comprehensive interpretation of the interactions between pathogens. We hope that future studies will improve upon this work by expanding geographic coverage and providing a more detailed assessment of the temporal dynamics of coinfections to validate and extend these findings.

## 5. Conclusions

Based on our current data, we recommend that patients undergo diagnostic testing for respiratory pathogens and that medical practitioners implement appropriate measures to control coinfections as early as possible. The timely and appropriate use of specific drugs, aided by accurate diagnosis, is essential for improving patient prognosis. Furthermore, new combined vaccines targeting multiple respiratory pathogens are important for intersecting epidemic prevention.

## Figures and Tables

**Table 1:** The distribution of coinfections of different respiratory pathogens in different age groups.

	COVID-Positive				Influenza-Positive				Other			
	<18	18–44	45–64	65+	<18	18–44	45–64	65+	<18	18–44	45–64	65+
Total number, N	42 (67.8%)	16 (25.8%)	3 (4.8%)	1 (1.6%)	2 (28.6)	5 (71.4)	0	0	13 (86.6%)	1 (6.7%)	1 (6.7%)	0
Testing												
SARS-CoV-2	-	-	-	-	0	2 (100%)	0	0	-	-	-	-
Influenza A virus	0	2 (100%)	0	0	-	-	-	-	-	-	-	-
*Haemophilus* *influenzae*	20 (71.4%)	7 (25.0%)	1 (3.6%)	0	1 (50.0%)	1 (50.0%)	0	0	8 (100%)	0	0	0
*Streptococcus* *pneumoniae*	15 (83.3%)	2 (11.1%)	0	1 (5.6%)	2 (66.7%)	1 (33.3%)	0	0	7 (87.5%)	1 (12.5%)	0	0
Rhinovirus	11 (78.6%)	3 (21.4%)	0	0	0	0	0	0	6 (85.7%)	0	1 (14.3%)	0
*Staphylococcus* *aureus*	14 (93.3%)	1 (6.7%)	0	0	0	0	0	0	3 (100%)	0	0	0
Respiratory syncytial virus	2 (100%)	0	0	0	0	0	0	0	3 (100%)	0	0	0
Coronavirus OC43	2 (66.7%)	0	1 (33.3%)	0	0	0	0	0	2 (100%)	0	0	0
Parainfluenza virus type I	1 (100%)	0	0	0	0	0	0	0	3 (100%)	0	0	0
Enterovirus	0	2 (100%)	0	0	0	0	0	0	1 (50.0%)	0	1 (50.0%)	0
Group A * Streptococcus *	1 (100%)	0	0	0	0	1 (100%)	0	0	1 (100%)	0	0	0
Adenovirus	2 (100%)	0	0	0	0	0	0	0	0	0	0	0
Parainfluenza virus type III	1 (50.0%)	0	1 (50.0%)	0	0	0	0	0	0	0	0	0
Klebsiella pneumoniae	0	1 (100%)	0	0	0	0	0	0	0	0	0	0
Human metapneumovirus	0	0	0	0	0	0	0	0	0	1	0	0
Parainfluenza virus type IV	1 (100%)	0	0	0	0	0	0	0	0	0	0	0

## Data Availability

The data supporting the findings of this study are available from the corresponding author upon reasonable request.

## Supplementary Materials

The following supporting information can be downloaded at https://www.mdpi.com/article/10.3390/pathogens13121113/s1, Table S1: SARS-CoV-2; Table S2: Other respiratory pathogens.
